# Exposure of US Travelers to Rabid Zebra, Kenya, 2011

**DOI:** 10.3201/eid1807.120081

**Published:** 2012-07

**Authors:** Emily W. Lankau, Joel M. Montgomery, Danielle M. Tack, Mark Obonyo, Samuel Kadivane, Jesse D. Blanton, Wences Arvelo, Emily S. Jentes, Nicole J. Cohen, Gary W. Brunette, Nina Marano, Charles E. Rupprecht

**Affiliations:** Centers for Disease Control and Prevention, Atlanta, Georgia, USA (E.W. Lankau, D.M. Tack, J.D. Blanton, E.S. Jentes, N.J. Cohen, G.W. Brunette, N. Marano, C.E. Rupprecht);; Centers for Disease Control and Prevention, Nairobi, Kenya (J.M. Montgomery, W. Arvelo);; and Ministry of Public Health and Sanitation, Nairobi (M. Obonyo, S. Kadivane)

**Keywords:** *Equidae*, Kenya, prevention and control, rabies, travel, world health, zebras, viruses, zoonoses

**To the Editor:** Rabies is an acute progressive encephalitis caused by infection with a lyssavirus (genus *Lyssavirus*, family *Rhabdoviridae*) (*1*). Most human infections are caused by bites from rabid animals, but the virus also can be transmitted by contact of open wounds or mucous membranes with animal saliva (*1**,**2*). Prompt administration of postexposure prophylaxis (PEP) is recommended to prevent rabies (*3*). Canids are common sources of human exposures in many regions of Africa, Asia, and Latin America (*4*). However, all mammals are susceptible, including herbivores such as horses, cattle, and antelope (*5**–**7*).

Approximately 16–200 rabies virus exposures occur per 100,000 international travelers (*2*). Travelers might be unaware of exposure risks from less commonly affected species because prevention guidelines focus on avoiding contact with feral and wild carnivores (primarily dogs) and bats (*2*). After travelers at a safari lodge in Kenya were exposed to a rabid zebra, the Centers for Disease Control and Prevention (CDC) and international partners conducted a contact investigation to ensure affected travelers received timely exposure assessments and appropriate PEP recommendations.

In January 2011, an orphaned zebra foal was taken to a safari lodge for care. Tourists were permitted to view, pet, and feed the zebra. A dog suspected of being rabid bit the zebra on July 31. Attempts to capture the dog for testing were unsuccessful. The zebra became ill around August 24 and died on August 26 (Figure).

**Figure:**
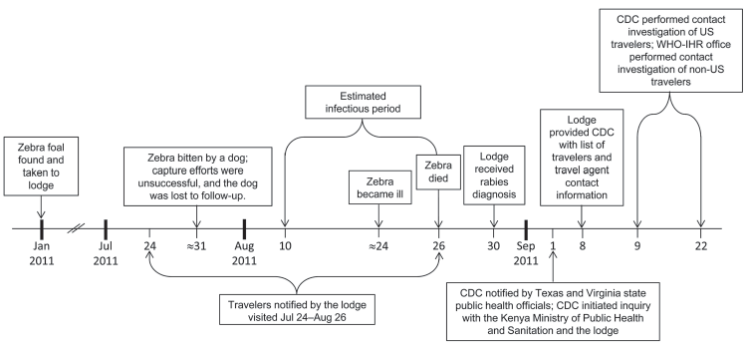
Timeline of events for traveler exposures to a rabid zebra and subsequent contact investigation of US travelers, Kenya, January 2011–September 2011. CDC, Centers for Disease Control and Prevention; WHO-HIR, World Health Organization’s International Health Regulations Office.

Rabies was suspected because of neurologic signs and was diagnosed in the zebra after detection of rabies virus antigens by direct fluorescent antibody testing at the Kenya Central Veterinary Laboratory. Lodge staff received results on August 30 and immediately communicated the information to travelers who had visited during July 24–August 26 by email through booking travel agents (because lodge staff did not have traveler contact information). This email conveyed the diagnosis and information about rabies virus transmission and vaccine and advised travelers to consult their physicians if they believed they were at risk.

On September 1, after receiving the email, several US travelers reported contact with the zebra’s mouth and saliva to state health officials. State health officials notified CDC that same day. CDC initiated a contact investigation of US travelers; the World Health Organization International Health Regulations Office coordinated contact investigation for non-US travelers. The Kenya Ministry of Public Health and Sanitation, Field Epidemiology and Laboratory Training Program, and the Kenya Wildlife Service performed environmental assessments, evaluated lodge staff and animal exposures, and reviewed bite surveillance and preparedness in the surrounding district. CDC Rabies Program staff corroborated the diagnosis and genotyped the variant as one associated with dogs in Africa, supporting the presumed transmission through dog bite.

On September 8, lodge staff provided CDC with travelers’ surnames, number of travelers per group, countries of citizenship, and residence, and travel agent contact information. Of 243 travelers, 136 (56%) were US residents from 14 states (Technical Appendix Table 1). The remaining 107 travelers were residents of 16 countries, primarily in Europe (Technical Appendix Figure). CDC obtained traveler contact information from travel agents. State health officials contacted US travelers by telephone or email.

Viral shedding duration for rabid zebras is unknown. An infectious period was estimated as the 14 days from the foal’s illness until its death (August 10–26) (*8*). Of 136 US travelers, 77 (57%) visited the lodge during this period. The remaining 59 US travelers who visited during July 24–August 9 also were contacted to document medical assistance received and to provide rabies education.

Twenty-eight (21%) US travelers had already initiated PEP when interviewed by state public health officials. Exposure risk categories based on Advisory Committee on Immunization Practices recommendations were developed to address the unique circumstances of this investigation, i.e., the period and nature of travelers’ exposures to the zebra (Technical Appendix Table 2) (*3*). None reported high-risk exposures; 2 reported moderate-risk exposures; and 26 reported low- or no-risk exposures, for which PEP would not have been recommended. CDC has not received any reports of human rabies in travelers exposed to the zebra in this incident.

Initial exposure notifications to travelers were delivered by travel agents, rather than public health officials. Public health intervention was delayed while traveler contact information was obtained. During this delay, travelers sought care from private physicians who made time-sensitive PEP decisions with incomplete information, resulting in unnecessary PEP administration according to published standards (*3*). Unnecessary PEP should be avoided because rabies biologicals are expensive (averaging $4,000/patient [*9*]), and rabies PEP entails small but real risk for adverse events (*3*). Inclusion of a health provision in travel agency privacy agreements to permit release of traveler contact information for public health use would improve response times for similar events.

Travelers to rabies-endemic regions should avoid contact with wild and feral animals, even in seemingly safe captive settings (*2*). Any mammal can be rabid, and infectious animals might appear healthy for several days before illness onset; avoiding all wild and feral animals while traveling is the ideal preventive measure. All animal bites and scratches should be washed thoroughly with soap and water and receive immediate medical attention (*2*).

## Supplementary Material

Technical Appendix Contact investigation, exposure risk categories, and contact investigation outcomes for US travelers, Kenya, July 24–August 26, 2011.
