# Supplementary material for: Exposure of US Travelers to Rabid Zebra, Kenya, 2011
Source: Emerg Infect Dis. 2012 Jul;18(7):1202–4. doi: 10.3201/eid1807.120081 (PMC3376810; doi:10.3201/eid1807.120081)
Supplement: Technical Appendix — Contact investigation, exposure risk categories, and contact investigation outcomes for US travelers, Kenya, July 24–August 26, 2011. [file 12-0081-Techapp_2p.pdf]

Article DOI: http://dx.doi.org/10.3201/eid1807.120081

Exposure of US Travelers to Rabid Zebra, Kenya, 2011

Technical Appendix

Technical Appendix Table 1. Contact investigation for US travelers exposed to a rabid zebra, Kenya, July 24–August 26, 2011

State of residence	Travel period Jul 24–Aug 9	Travel period Aug 10–26		Total
		Contact*	No contact	
California	3	12	13	28
Texas	11	2	2	15
New York	1	3	9	13
Maryland	9	3	0	12
Washington	0	5	6	11
New Jersey	6	4	0	10
Virginia	0	3	5	8
Florida	2	0	5	7
Pennsylvania	7	0	0	7
Colorado	4	1	0	5
Connecticut	5	0	0	5
Illinois	5	0	0	5
Indiana	2	0	0	2
Utah	2	0	0	2
Unknown	2	0	4	6
Total	59	33	44	136

*Direct physical contact with the zebra as self-reported by travelers.

Technical Appendix Table 2. Exposure risk categories for travelers exposed to a rabid zebra, Kenya, July 24–August 26, 2011

Exposure category	Period of contact	Description
High risk	Aug 10–26*	Traveler reported being bitten† by the zebra or having fresh, wet zebra saliva in direct contact with open skin wounds or mucous membranes
Moderate risk	Aug 10–26*	Traveler reported having skin contact with fresh, wet zebra saliva and reported the possibility of skin wounds at contact.
Low risk	Aug 10–26*	Traveler reported having touched or fed the zebra but no contact between fresh, wet saliva and open wounds or mucous membranes.
No risk	Jul 24–Aug 9	Contact with the zebra occurred before the infectious period.

*Estimated infectious period.
†None of the US travelers contacted reported being bitten by the zebra.

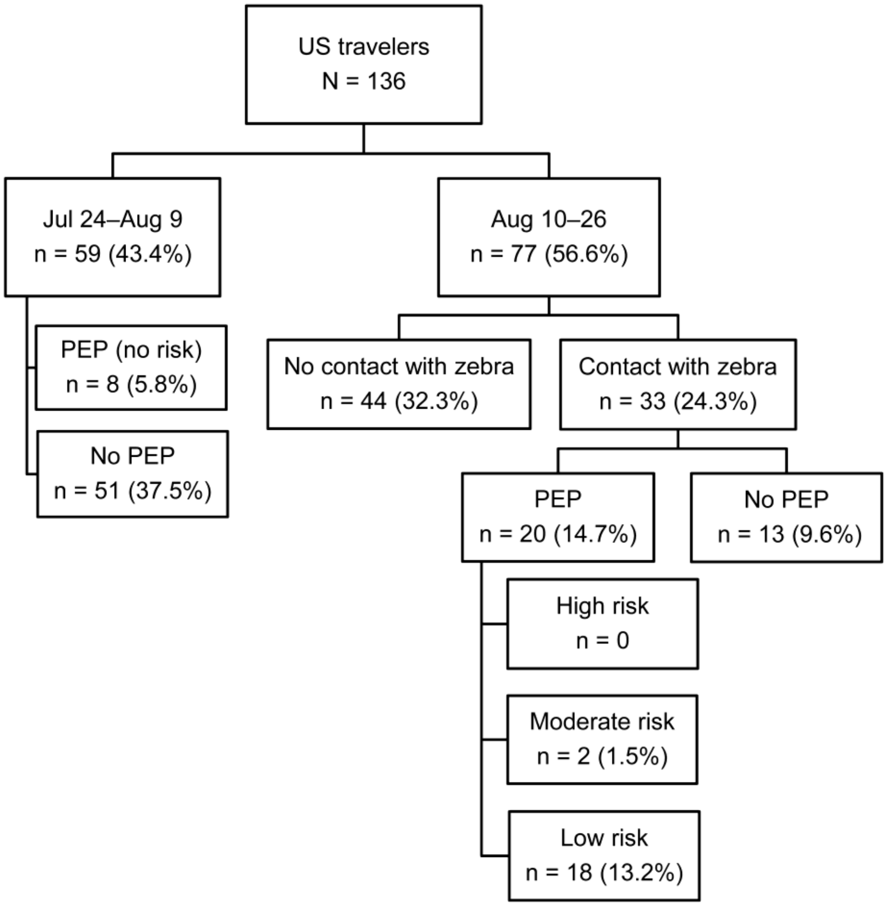

Technical Appendix Figure. Contact investigation outcomes for 136 US travelers, by travel period and postexposure prophylaxis (PEP) outcomes, Kenya, July 24–August 26, 2011. Exposure risk categories are defined in Technical Appendix Table 2.
